# 

**Published:** 1921-10

**Authors:** 


### ANNOUNCEMENTS

### MICHIGAN DENTAL EXAMINATION

The next examination for candidates wishing to practice dentistry in Michigan will be held in Ann Arbor during the week beginning November 14th, 1921. All credentials and fees must be in the hands of the Secretary not later than November 4th. For application blanks write to E. O. Gillespie, 745 David Whitney Bldg., Detroit, Michigan.—E. O. GILLESPIE, *Secy.-Treas.*

### THE AMERICAN COLLEGE OF DENTISTS

There has recently been organized within the profession in the United States an honorary organization whose membership will constitute a roll of honor in dentistry. It is the aim and purpose of this organization to enroll only such names as represent genuine merit, through conscientious effort or noble and deserving sacrifice within the ranks of the dental profession. The entire sphere of intention of this organization is one of uplift of professional spirit, conduct and responsibility.

Organization has been brought about through a recognition of the need of some strong influence along these lines, and it is through the Dental Editors club that the official statement, which follows, of its organization and objects is given to the profession.

During the annual meeting of the N. D. A. the Dental Editors club presented a resolution to the Board of Trustees relative to the appointment by the National Dental Association of a committee on Nomenclature. It is gratifying to learn that the board not only approved the resolution but referred it to the House of Delegates, recommending its passage, which resulted in favorable action and the following committee appointed:

L. P. Anthony, chairman, Philadelphia; C. N. Johnson, vice chairman, Chicago; Otto U. King, secretary, Chicago; H. E. Friesell, Pittsburgh; Herbert L. Wheeler, New York.

*Statement of Objects and Requirements for Fellowship*

Every important profession, science or art has its Academy, Legion, or Court of Honor, to which are elected, or appointed, those who have unselfishly devoted themselves to the advancement of each specific cause. This has been done not only as a just recognition of meritorius services, but also as an example to younger members that they may be encouraged to nobler efforts.

Recognition of the need of a similar influence in dentistry has resulted in the establishment of the American College of Dentists. The object of this College is to bring together in a group the men of outstanding prominence in the profession and by their united efforts in a field that is not now covered by any dental agency to endeavor to aid in the advancement of the standards and efficiency of American Dentistry. Some of the aims of the College are to cultivate and encourage the development of a higher type of profession spirit and a keener sense of social responsibility throughout the profession; by precept and example to inculcate higher ideals among the younger element of the profession, and hold forth its Fellowship as a reward to those who faithfully follow such ideals; to stimulate advanced work in dental art, science and literature; and to honor men who have made notable contributions to the advancement of our profession.

The enormously increased responsibilities of the dental profession to humanity on the one hand; the unpresented opportunities for exploitation, which have resulted in a wave of mercenary practices that threatens to become a public scandal to the everlasting disgrace of American Dentistry, on the other hand, demand that those elements of the profession, whose character, reputation and professional attainments point them out as leaders, should be brought together for the purpose of checking the tide of destructive agencies and of encouraging by every laudable means the cultivation of that high spirit of professional and social responsibility, the wholesome influence of which is so greatly needed.

Inasmuch as there is no title or mark of distinction to differentiate the recent graduates from the practitioner who has devoted many years of faithful effort in the upbuilding of his profession, it is proposed that the Fellowship of the College shall be conferred upon two groups of practitioners, viz.:

1. Upon those members of the profession who have been at least ten years engaged in the practice of dentistry, whose efforts during that time have been loyally devoted to its advancement, and who are unquestionably looked upon as leaders in the respective communities. Time and effort devoted to teaching in dental schools, to presenting papers, or clinics before dental societies, or to organization and executive work of a constructive character; as well as public services or civic duties having a tendency to enlarge the usefulness or the public appreciation of dentistry, shall be taken into consideration when passing upon candidates of this group.

2. The conferring of the Fellowship shall be held out as a stimulus to young men to induce them to engage more earnestly in those activities which tend to advance dentistry as a profession and for which monetary remuneration must necessarily be sadly out of proportion to the time and effort expended. Devotion to teaching, especially in the non-clinical branches; to research work and to public education, as well as advanced work in the art, science or literature of dentistry, should be greatly encouraged as a consequence of this movement.

The candidate for the Fellowship in either class must be of good moral character, and have a reputation for ethical conduct and professional standing that is unquestioned. Personality, integrity, education unselfishness, and high professional ideals, as well as freedom from mercenary tendencies, shall be considered in evaluating the qualifications of all candidates for the Fellowship.

---

### CHICAGO DENTAL SOCIETY'S ANNUAL CLINIC

The annual clinic of the Chicago Dental Society will be held at the magnificent new Hotel Drake, January 19, 20, 21, 1922.

This hotel is peculiarly well adapted to the needs of a large sectional meeting as has been planned for this occasion. Many commodious sound-proof rooms will be provided for the lectures and demonstrations, while the space for the general sessions and exhibits will meet all requirements, and is ideal in every particular.

The program, which will be announced at a later date, will surpass anything yet attempted by this component society.

For convenience, out of town guests should make their reservations at the Drake,, and they should be made as early as possible, as there will be a large attendance from outside Chicago. All ethical dentists are cordially invited.

Further information will be cheerfully given by addressing M. M. Printz, secretary, 25 E. Washington Street, Chicago.

---

### NOVEMBER SELECTED AS "PERFECT PACKAGE MONTH" BY THE NATION'S CARRIERS

All trades and industries have been asked to co-operate in the "Perfect Package Movement" to be inaugurated by the railroads, steamship lines and express companies in the United States and Canada, in November, which has been designated as "Perfect Package Month."

The purpose of the movement is to stimulate further public interest in good packing of shipments and to enable the carriers to improve the transportation service of the country. During November, an examination of all shipments sent by freight or express, will be conducted, to obtain information as to the best shipping methods carried on by the various trades and industries.

In every city and town, the railroad and express people will form campaign committees, to co-operate with local shippers' associations, in carrying out the plans announced for "Perfect Package Month." "Exception Reports" will be made out for all faulty shipments discovered and these reports will be sent to the shippers' association for tabulation, to ascertain how high a percentage for "Perfect Packages," the shippers of that city have attained.

Comparisons of the records made by the various cities during November will be announced at the conclusion of the drive. The entire working forces of the railroad and express carriers, comprising some 2,000,000 men, will aid in the movement. The railroads, through the American Railway Association, composed of practically all of the railroads in the country, are pushing the campaign, as a means of raising the standard of the service, while the express agents are also getting ready to interest shippers in the undertaking.

---

A Berlin Dental Clinic Closed. On June 1st the dental clinic of the Berlin Institute for Rural Administration was finally closed. The technical department came to an end some time ago. The high cost of upkeep is the principle reason for the action of the authorities in this matter. A report of work done since 1914 has recently been issued from the Institute. The number of dentures supplied in that year was 3,933; in 1915, 2, 732; in 1916, 2,723; in 1917, 1,549; in 1918, 2,935; and in 1919, 4,095.—*Zahnaerztliche Rundeschau.*

IN subscribing to the fund of the Dental Welfare Foundation, be sure to avoid imposters who do not represent the Dental Welfare Foundation through the accredited channels of all the dental depots in the United States and Canada. Be sure the agent soliciting your subscription is from your local depot. You will most likely know him personally.

This warning is posted to place the Dental Profession on its guard against imposters and confidence men.

